# Supplementary material for: RNA aptamer reveals nuclear TDP-43 pathology is an early aggregation event that coincides with STMN-2 cryptic splicing and precedes clinical manifestation in ALS
Source: Acta Neuropathol. 2024 Mar 5;147(1):50. doi: 10.1007/s00401-024-02705-1 (PMC10914926; doi:10.1007/s00401-024-02705-1)
Supplement: Supplementary file 1 — Supplementary file1 (DOCX 2513 kb) [file 401_2024_2705_MOESM1_ESM.docx]

**Supplementary Information**

**Article title:**

RNA aptamer reveals nuclear TDP-43 pathology is an early aggregation event that coincides with *STMN-2* cryptic splicing and precedes clinical manifestation in ALS

**Journal name:** Acta Neuropathologica

**Author names:**

Holly Spence^1¥^, Fergal M. Waldron^1¥^, Rebecca S. Saleeb^2,3^, Anna-Leigh Brown^4^, Olivia M. Rifai^5^, Martina Gilodi^6^, Fiona Read^1^, Kristine Roberts^7^, Gillian Milne^1^, Debbie Wilkinson^1^, Judi O’Shaughnessy^2,3^, Annalisa Pastore^8^, Pietro Fratta^4^, Neil Shneider^9^, Gian Gaetano Tartaglia^6^, Elsa Zacco^6^, Mathew H. Horrocks^2,3^*, Jenna M. Gregory^1,7^*.

^¥^*Denotes equal contribution.

**Affiliations:**

^1^Institute of Medical Sciences, University of Aberdeen, Aberdeen, UK.

^2^EaStCHEM School of Chemistry, University of Edinburgh, Edinburgh, UK.

^3^IRR Chemistry Hub, Institute for Regeneration and Repair, University of Edinburgh, Edinburgh, UK.

^4^Department of Neuromuscular Diseases, UCL Queen Square Institute of Neurology, London, UK.

^5^Centre for Discovery Brain Sciences, University of Edinburgh, UK.

^6^RNA System Biology Lab, Instituto Italiano di Tecnologia, Genoa, Italy.

^7^NHS Grampian tissue biorepository, Department of Pathology, Aberdeen, UK.

^8^The Maurice Wohl Institute, King's College London, London, UK.

^9^Department of Neurology, Center for Motor Neuron Biology and Disease, Columbia University, New York, USA.

***Correspondence to:** [jenna.gregory@abdn.ac.uk](mailto:jenna.gregory@abdn.ac.uk) or [mathew.horrocks@ed.ac.uk](mailto:mathew.horrocks@ed.ac.uk)

**Supplementary Figures 1-4:**


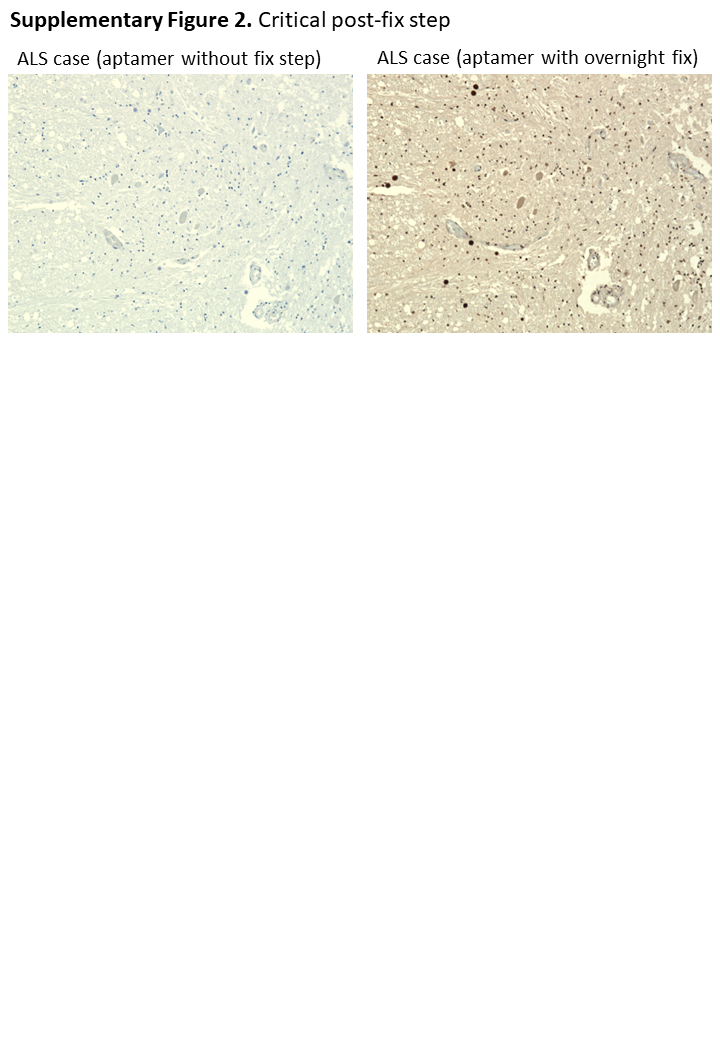
**Supplementary Figure 1. Critical Post-Fix Step and RNA negative control.**

Serial sections from the spinal cord of a patient with sporadic ALS stained without (left image) and with (right image) the critical post-fix step (incubation with 4% PFA overnight after aptamer incubation) demonstrate that it is necessary to immobilise the aptamer on to the target using formalin cross links to enable immunodetection. Scale bar is 200 µM.

**
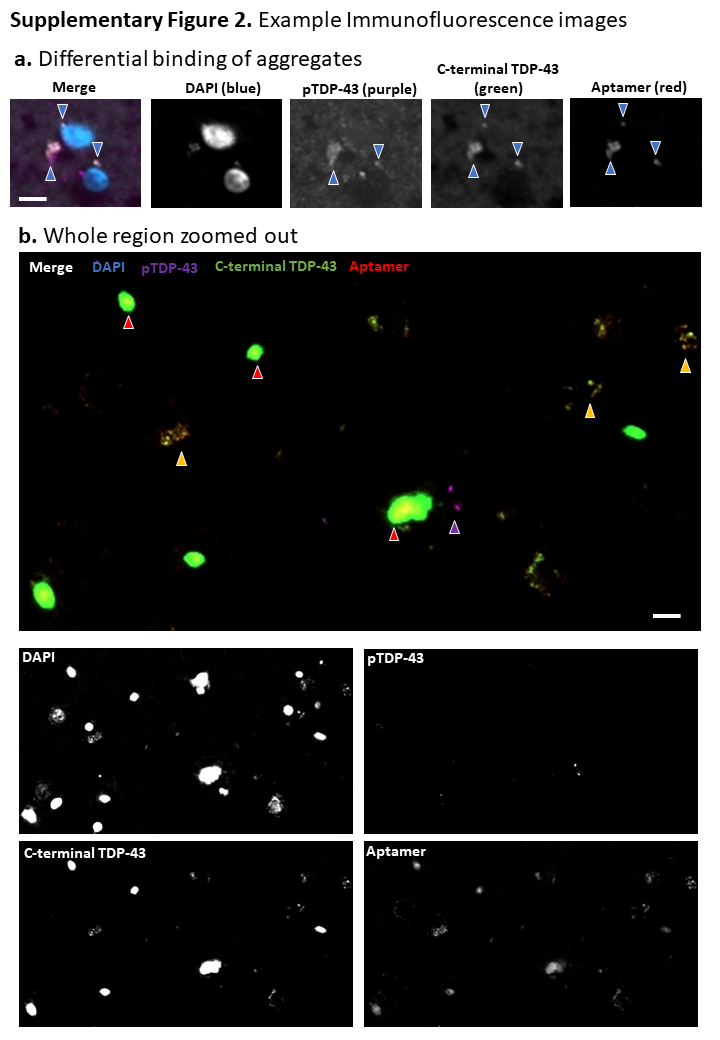
**

**Supplementary Figure 2. Immunofluorescence images demonstrating differential overlap between TDP-43 antibodies and TDP-43^APT^ staining patterns.**

Immunofluorescence images showing **a.** Two neuronal cells containing cytoplasmic TDP-43 aggregates that stain for all three antibody markers (pTDP-43 in purple, c-terminal TDP-43 in green and TDP-43^APT^ in red). Blue arrows indicate an aggregate present in all three channels. Scale bar is 5 µm. **b**. Zoomed out region with extensive TDP-43 aggregation - immunofluorescent image demonstrating the variation in aggregation events, with evidence of pTDP-43 immunoreactive aggregates (purple arrowhead) alone as well as co-incident immunoreactivity in some aggregates for c-terminal antibody and TDP-43^APT^ (yellow arrowhead). There are also multiple examples of TDP-43^APT^ staining within the nucleus which is obscured by normal c-terminal TDP-43 antibody staining. Scale bar is 10 µm.

**
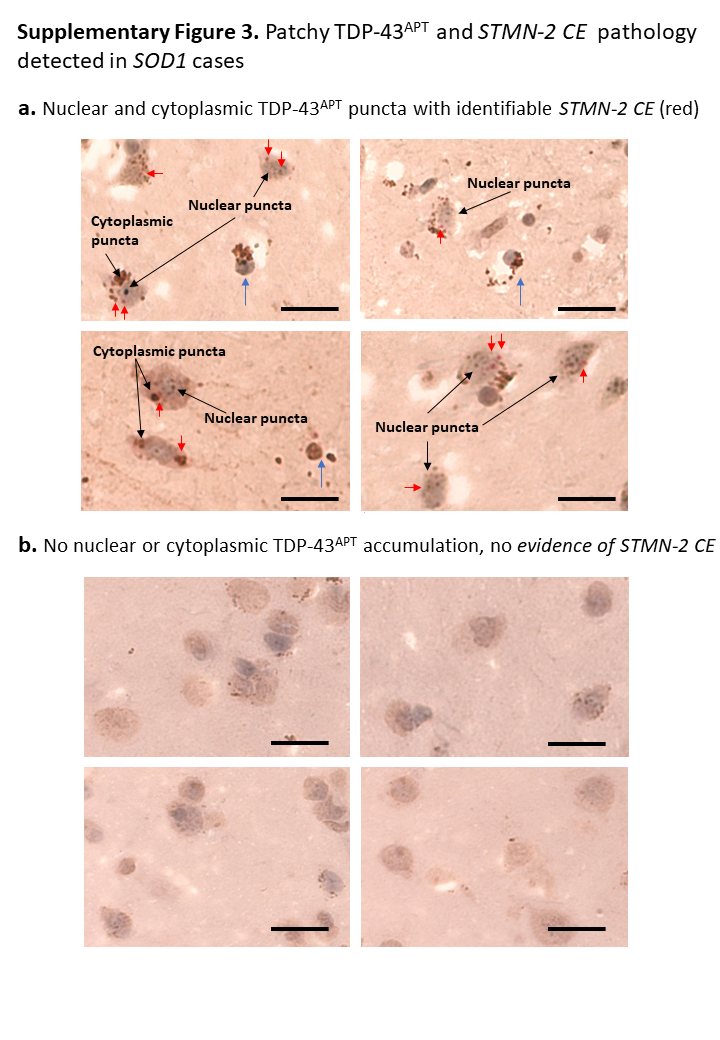
Supplementary Figure 3. Patchy TDP-43^APT^ and *STMN-2 CE* pathology detected in *SOD1* cases.**

Representative photomicrographs taken at 40x magnification demonstrating dual DAB immunohistochemical staining for TDP-43^APT^ and *in situ* hybridization with BaseScope^TM^ to detect *STMN-2* cryptic exon (CE) mRNA transcripts (individual red dots are single mRNA transcripts of *STMN-2* and are indicated by red arrows). TDP-43 pathology (both TDP-43^APT^ and ^STMN-2 CE^ are present in *SOD1* cases, but the distribution is patchy, with areas demonstrating both TDP-43^APT^ and *STMN-2 CE* pathologies (**a.**) and other areas showing no evidence of either pathology (**b.**) Images show the presence of cryptic exons (red arrows) in neurons with nuclear and cytoplasmic TDP-43^APT^ puncta (brown staining). Glial pathology can also be seen in the background (blue arrows). Sections are counterstained with haematoxylin. Scale bar is 20 µm.

**
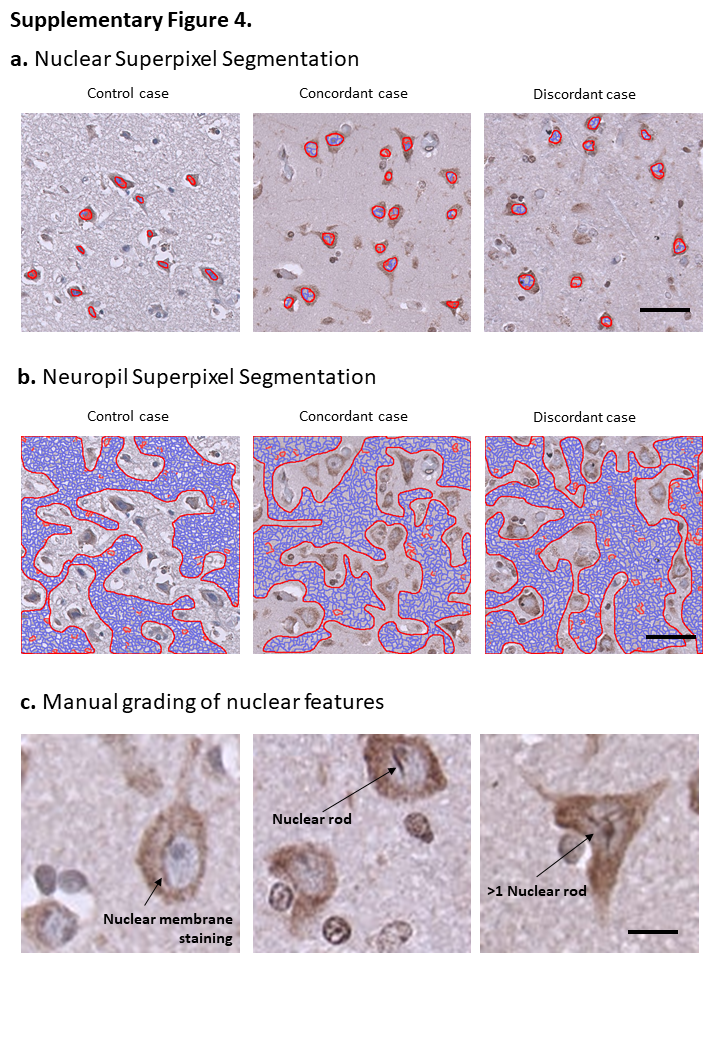
**

**Supplementary Figure 4.**

Example images of nuclear superpixel segmentation (**a**) and neuropil superpixel segmentation (**b**) performed using QuPath software and example images of nuclear features graded by blinded manual assessment (**c**).
